# Polystyrene*-co-*Divinylbenzene PolyHIPE Monoliths in 1.0 mm Column Formats for Liquid Chromatography

**DOI:** 10.3390/ma9030212

**Published:** 2016-03-18

**Authors:** Sidratul Choudhury, Laurence Fitzhenry, Blánaid White, Damian Connolly

**Affiliations:** 1 National Centre for Sensor Research, School of Chemical Sciences, Dublin City University, Dublin 9, Ireland; disharani30@gmail.com; 2 Pharmaceutical and Molecular Biotechnology Research Centre (PMBRC), Department of Science, Waterford Institute of Technology, Waterford, Ireland; l.fitzhenry@wit.ie

**Keywords:** polystyrene, polyHIPE, silcosteel, microbore, reversed phase LC, isocratic separation

## Abstract

The reversed phase liquid chromatographic (RP-HPLC) separation of small molecules using a polystyrene-*co*-divinylbenzene (PS*-co-*DVB) polyHIPE stationary phases housed within 1.0 mm i.d. silcosteel columns is presented within this study. A 90% PS*-co-*DVB polyHIPE was covalently attached to the walls of the column housing by prior wall modification with 3-(trimethoxysilyl) propyl methacrylate and could withstand operating backpressures in excess of 200 bar at a flow rate of 1.2 mL/min. Permeability studies revealed that the monolith swelled slightly in 100% acetonitrile relative to 100% water but could nevertheless be used to separate five alkylbenzenes using a flow rate of 40 µL/min (linear velocity: 0.57 mm/s). Remarkable column-to-column reproducibility is shown with retention factor variation between 2.6% and 6.1% for two separately prepared columns.

## 1. Introduction

Monoliths are a single continuous rod of highly porous material polymerized *in situ* within a mould with an interconnected network of pores that form channels through which liquids can flow. Monoliths have found a wide range of uses in analytical science such as their incorporation into microfluidic platforms [1], high efficiency chromatographic separations or low resolution *“digital chromatography”* (bind and elute methods) in the form of solid phase extraction. The reader is directed to a number of excellent reviews that have appeared in the literature [2,3], particularly some reviews which specifically focus on polymer monoliths for the separation of small molecules [4,5,6,7,8].

Monoliths are broadly subdivided between inorganic (mainly silica) and organic monoliths, the latter having previously been considered more suited to the gradient separation of large biomolecules rather than the isocratic separation of smaller (<500 Da) molecules due to their relatively low surface area (<50 m^2^/g). However, it has been shown that low surface area is not the sole factor that limits chromatographic efficiency for small molecules. Rather, studies by Nishang *et al.* [8,9,10,11] have revealed that maximizing chromatographic efficiency under equilibrium (isocratic) conditions actually relies upon a more complex strategy involving the combined optimization of both the surface area/microscopic morphological properties as well as the nanoscale gel porosity (which can induce stagnant mass transfer zones, thus increasing resistance to mass transfer within the porous, globule-scale polymer matrix). Nischang [11] and Causon *et al.* [12] have demonstrated that the gel porosity of the polymer scaffold can be optimised for small molecules depending upon the percentage of organic modifier in the mobile phase. Furthermore, the gel porosity is absent in the monolith’s dry-state such that dry-state monolith characterisation methods alone (nitrogen adsorption analysis or scanning electron microscopy) are insufficient to fully explain the chromatographic performance of polymer monoliths [11]. Laher *et al.* demonstrated the benefits of alternative methods with commercial styrene and methacrylate monoliths such as confocal Raman spectroscopy imaging and atomic force microscopy [13]. 

Notable successful efforts to improve chromatographic performance include stopping monolith polymerisation reactions before completion. Nischang *et al.* [14] expanded upon earlier work by Bonn’s group [15,16] by demonstrating that the surface area of styrenic monoliths could be increased from 5–6 m^2^/g to 100 m^2^/g by reducing the polymerisation time from 48 h to 3 h, respectively. Completely polymerized material (48 h) exhibited high peak widths and increased retention relative to the three hour material, and the authors observed that the enhanced performance for monoliths derived from incomplete conversion was less affected by specific surface area, rather than the gel porosity of the solvated polymer globules. Similar observations were made for methacrylate-based monoliths subjected to incomplete polymerisation [17], reducing the polymerisation time from 48 h to 30 min, which resulted in decreased retention for alkylbenzenes and significantly decreased plate heights, despite relatively low BET surface area of 5.85 m^2^/g (30 min polymerisation). Another notable example of relatively low surface area monoliths exhibiting high chromatographic efficiency was reported recently by the Zuo group [18], who used a cross-linker with multiple acrylate groups (dipentaerythritol penta-/hexa-acrylate, DPEPA) to prepare a poly(lauryl methacrylate*-co-*DPEPA) monolith for the isocratic separation of alkylbenzenes resulting in 110,000-165,000 N/m. The surface area of this monolith was comparatively low at 27.4 m^2^/g, but the authors concluded that the enhanced performance was actually due to the use of the multi-functional crosslinker which prevented the generation of gel-like micropores in the monolith globules which otherwise would result in high *C*-term values. An alternative strategy to increase nanoscale gel porosity is hypercrosslinking whereby poly(styrene-covinylbenzylchloride-*co*-divinylbenzene) monoliths are swollen in dichloroethane followed by Friedel–Crafts reaction with ferric chloride as catalyst at a temperature of 80 °C resulting in surfaces areas of several hundred m^2^/g and chromatographic efficiency >75,000 N/m [19,20]. Monoliths modified with selected nanoparticles have also resulted in impressive chromatographic performance as detailed in several reviews on the subject [21,22,23,24].

Traditional polymeric monoliths are formed by using a mixture of functional monomers, crosslinker, thermal or photo-initiators and appropriate porogen(s), leading to a traditional “cauliflower-like” morphology. A more recent method of polymer monolith fabrication is high internal phase emulsion (HIPE) templating. When a HIPE is formed, one phase is dispersed within the other in spherical droplet form, forming the “internal phase” and must be greater than 74% by volume [25]. In a water-in-oil (W/O) emulsion the water acts as the porogen and is dispersed in the organic phase (“continuous phase”) which contains polymerisable monomers. This internal phase fraction (water) is known as the discontinuous phase since it is removed after polymerisation of the polyHIPE [25,26]. The spherical macropores present due to the removal of porogen are known as voids (typically up to 10 µm in size), and the interconnecting pores in the walls of the voids are referred to as windows (usually 1–2 µm) [25]. 

The use of polyHIPEs in separation science has recently been reviewed [27] and is restricted mainly to the use of the crushed/powdered polyHIPEs for the extraction of analytes in batch mode, or their fabrication in flow through formats for low-efficiency trap-and-release cleanup of biological samples. Only very few examples of polyHIPEs for the separation of small molecules (<1000 Da) are reported and are worthy of discussion here. In 2010, Tunc *et al.* fabricated a 90% porosity isodecylacrylate*-co-*divinylbenzene polyHIPE in 100 µm i.d. fused silica capillary and separated alkylbenzenes, yielding efficiency values in excess of 200,000 N/m when the monolith was operated in capillary electrochromatography (CEC) mode [28]. Later, in 2012, the same group investigated the application of PS*-co-*DVB polyHIPEs in CEC but reported a somewhat lower resolution and separation efficiency relative to their earlier work [29]. Recently, Hughes *et al.* formed PS*-co-*DVB polyHIPEs and EDMA polyHIPEs in 4.6 mm i.d. stainless steel column housings for the chromatographic separation of metal nanoparticles [30]. The monoliths were not bonded to the column wall and although 5 nm and 10 nm gold nanoparticles could be separated, peak symmetry factors exceeded 2.2 for each peak, and a very poor resolution of only 0.31 was achieved. 

When polymer monoliths have been used in separation science, either for liquid chromatographic or electrophoretic separations, the monolith has typically been formed within fused silica capillary or within the confines of a channel on a microfluidic chip (usually cyclic olefin co-polymer) [1]. In all cases, any useful monolith housing must obey two important criteria. Firstly, the diameter of the column/channel is usually <300 µm because the heat generated during monolith polymerization is not easily dissipated when larger diameters are used, typically resulting in poor radial homogeneity of monolith density. The confinement effect of the capillary wall due to the decreased surface-to-volume ratio, and strategies used to overcome this effect have been reported by He *et al.* [31] and Nischang *et al.* [32,33]. Furthermore, Mullner *et al.* have used serial block-face scanning electron microscopy to study the morphology of polymer monoliths formed within capillaries and demonstrated that macroporosity increased from the wall to the centre [34]. Secondly, the inner surface of the housing should be readily modified to facilitate covalent attachment of the resulting polymer monolith, thus eliminating a void at the wall and permitting the monolith to be operated at reasonable backpressures (at least 200 bar), without unwanted extrusion of the monolith from its housing. The former requirement limits the chromatographer to use specialist chromatographic instrumentation capable of pumping accurately at flow rates <50 µL/min, while minimizing both extra-column band-broadening as well as system dwell volumes if gradient separations are required. The advantage of using column housings between 500 µm and ~1.0 mm i.d. include the use of standard instrumentation usually intended for micro-bore or standard-bore liquid chromatography, with only a minor adjustment in plumbing/tubing configurations to maintain chromatographic efficiency. 

In this paper, we have for the first time applied a PS*-co-*DVB polyHIPE to the isocratic separation of small molecules under pressure-driven (rather then electro-osmotic) flow. Furthermore, the secure covalent attachment of a polyHIPE within a 1.0 mm i.d. microbore steel column is also demonstrated, a housing that has not previously been employed with polyHIPE materials. Finally, the chromatographic performance of these monoliths were evaluated using pressure and permeability measurements as well as the chromatographic separation of selected alkylbenzenes in the isocratic mode.

## 2. Results and Discussion

### 2.1. Polymerisation of Glycidyl Methacrylate PolyHIPES in Poly Etherether Ketone (PEEK) Tubing

**Figure 1:**
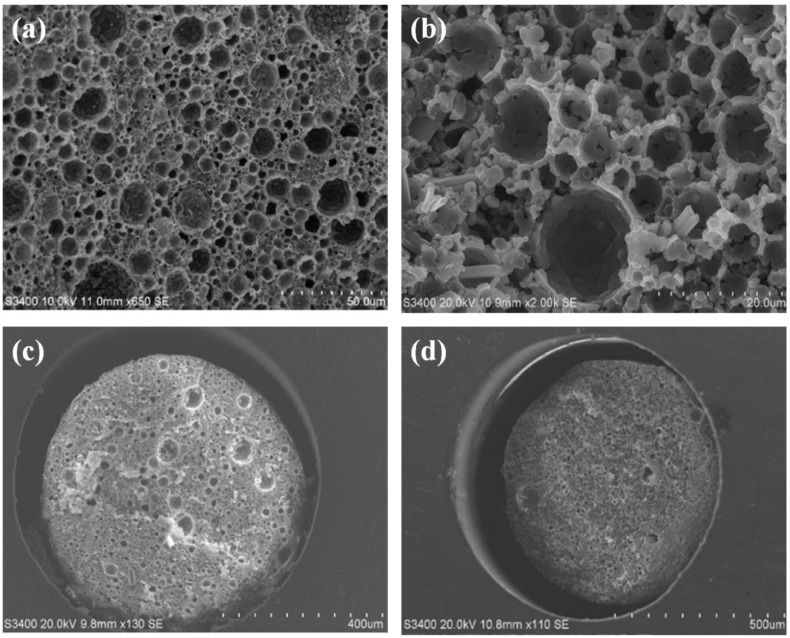
GMA*-co-*EDMA polyHIPE with (**a**) 4.5% surfactant; and (**b**) 5.5% surfactant. A GMA*-co-*EDMA polyHIPE with 4.5% surfactant was formed in (**c**) unmodified 0.76 mm i.d. PEEK; and (**d**) GMA-modified 0.76 mm i.d. PEEK.

Initial efforts to fabricate a polyHIPE in a microbore format were based on the work of Shu *et al.* who reported the fabrication of a lauryl methacrylate monolith in 1.0 mm i.d. PEEK tubing for the reversed phase separation of alkylbenzenes [35]. This involved the initial addition of methacryloyl groups to the inner surface of the PEEK to facilitate covalent attachment of the monolith to the column housing. Although Shu *et al.* worked with a reversed phase monolith, in this work we elected in the first instance to prepare a GMA*-co-*EDMA polyHIPE using an emulsion formulation described by Kranjc *et al.* [36]. The main advantage of GMA as functional monomer is that the pendant epoxy groups on the pore surface render this monolith reactive [37] such that the surface chemistry and thus chromatographic selectivity can be tailored to a particular application; indeed, Krajnc *et al.* reported the facile functionalisation of GMA*-co-*EDMA polyHIPEs formed within CIM disk housings (3 mm × 12 mm i.d.) with diethylamine for the ion-exchange separation of selected proteins [36]. The authors prepared a range of GMA*-co-*EDMA polyHIPEs wherein the aqueous phase volume % was varied during emulsion preparation (60%, 75%, 80% and 90%) and they reported corresponding increases in average pore size. However, their monoliths also exhibited a very wide distribution of void sizes, with several large crater-like pores present (~10 times larger than the average void size), which increased both in size and number as the aqueous phase volume % increased. In our hands, the same phenomenon was noted, and so efforts to eliminate this effect by variation of aqueous phase volume % were deemed redundant. Instead, different surfactant concentrations were trialed during emulsion preparation but again the resulting polyHIPEs were punctuated by several unusually large craters amongst a population of smaller voids as shown in Figure 1a,b which represent surfactant concentrations of 4.5 %v/v and 5.5 %v/v, respectively. For example, the average +/− pore size was 7.391 ± 6.727 µm (*n* = 30) for Figure 1a. 

Our overarching goal was to demonstrate the use of polyHIPEs for isocratic separations of small molecules. This requires a tighter size distribution of void sizes for improved chromatographic efficiency, as previously reported by Nischang [11], Causon *et al.* [38] and Gritti *et al.* [39] who studied the separation efficiency for small retained molecules on commercial polymer monoliths. For this reason, GMA*-co-*EDMA polyHIPES were rejected as candidates for further study, but not before also investigating the possibility of forming the polyHIPES within a PEEK column housing (albeit with non-optimum pore morphology). To this end, a comparison was made by forming a GMA*-co-*EDMA polyHIPE within unmodified PEEK (Figure 1c) and within PEEK that was surface modified with glycidyl methacrylate (Figure 1d). Unsurprisingly, large sections of the monolith within the unmodified tubing were easily pushed out by a modest flow of solvent but this was also the case for the modified tubing; in both cases, a large wall void is clearly seen and the remaining polyHIPE appeared to have shrunken in size within its mould. It is interesting to note that no other reports appear in the literature in which the work of Shu *et al.* [35] has successfully been repeated. However, during the preparation of this manuscript, a report has recently emerged in the literature in which the combination of sodium and vanadium(V) as catalysts during the PEEK sulphonation step significantly increases the yield of surface anchoring ligands [40], and this technique will be the subject of further research within our group. The remainder of our studies therefore involved the use of alternative column housings with more readily modifiable surface chemistry. Although stainless steel tubing is available in a range of geometries and has been used for monoliths before [41], it is not readily functionalised with anchoring groups due to the poor hydrolytic stability of the Me-O-Si-C bonding. Glass-lined steel tubing was considered but not pursued since it is difficult to cut to required lengths without shattering the glass lining; similarly, titanium housings [42,43] were not considered since their use requires a lengthy oxidation step in a furnace (6–8 h). Conversely, *“silcosteel”* tubing is commercially available in semi-microbore (1.0 mm) and microbore diameters (2.2 mm) and is lined with SiO_2_ such that the inner surface can readily be modified with double bonds using a reactive silane (3-(trimethoxysilyl) propyl methacrylate), to facilitate covalent attachment of the polyHIPE monolith. This tubing material was used for the first time in 2006 by Umemura *et al.* [44], who fabricated a 200 mm × 1.0 mm i.d. hexyl methacrylate*-co-*EDMA monolith for the gradient separation of four proteins within 20 seconds. Later in 2011, Shu *et al.* produced a 100 mm × 1.0 mm i.d. lauryl methacrylate*-co-*EDMA monolith in silcosteel tubing and separated 10 proteins using a two-minute gradient method [45].

### 2.2. Polymerisation of Polystyrene-co-Divinylbenzene PolyHIPEs in Silcosteel Tubing

Before fabricating a PS*-co-*DVB polyHIPE in silcosteel tubing (this monolith being likely to have a more permeable pore morphology relative to the GMA*-co-*EDMA polyHIPE), some preliminary experiments were first conducted to investigate the effect of aqueous phase ratios upon pore size and surface area. Therefore, 75%, 80% and 90% PS*-co-*DVB bulk polyHIPEs were produced and characterised using scanning electron microscopy (SEM) and Brunauer–Emmett–Teller(BET) surface area analysis. As expected, when the aqueous phase percentage was decreased, the pore morphology also changed. Specifically, the number of interconnecting windows between the larger voids decreased when the aqueous phase percentage was decreased, and this finding is in keeping with reports from the Cameron group [25,46,47]. Furthermore, as the aqueous phase ratio decreased, the voids also decreased in size from 12.9 ± 1.9 µm for the 90% polyHIPE, to 10.4 ± 2.5 µm for the 80% polyHIPE and 7.5 ± 1.0 µm for the 75% polyHIPE (*n* = 30 in each case), again in keeping with literature reports [25,46,47]. This represents a 42% decrease in average pore size between the two extremes of porosity under investigation in this study and would be expected to result in an increase in surface area. Indeed, surface areas were 20.1 m^2^·g^−1^, 24.3 m^2^·g^−1^ and 23.3 m^2^·g^−1^ for the 90%, 80% and 75% polyHIPEs respectively, corresponding to a 16% increase in surface area from the 90% polyHIPE to the 75% polyHIPE; these surface areas and the general increasing trend are typical for this type of polyHIPE [29]. Since the increase in surface area for the lower porosity monolith was only slight, the 90% polyHIPE formulation was selected for further studies in silcosteel column formats due to the anticipated higher permeability because of the larger flow-through void sizes.

A larger diameter silcosteel tube (2.2 mm i.d.) was first trialled with the 90% polyHIPE formulation, but no monolith formed at 60 °C. Even after 48 h of incubation time, the still liquefied emulsion was easily extruded from the column with a solvent flush, and after increasing the polymerisation temperature to 75 °C, a glass-like polymer adhered to the inner walls only, leaving a large gap through the central bore of the column. For this reason, the use of 2.2 mm i.d. housings was rejected in favour of 1.0 mm i.d. tubing. It should be noted that polyHIPEs have been fabricated in housings as large as 11 mm i.d. [48], however these reports are for oligo(ethylene-glycol)methacrylate*-co-*GMA*-co-*EDMA polyHIPEs with an obviously very different formulation than the one described herein, and the polyHIPE housing was a glass vial rather than a metal housing. Nesterenko *et al.* formed methacrylate monoliths in a 0.8 mm i.d. titanium housing and were forced to utilize a complex temperature gradient during polymerisation to minimize the occurance of a radial gradient of monolith density across the column diameter [42]. This counteracted the inability of the polymerisation system to effectively dissipate the heat of polymerisation, a phenomenon reported as early as 1997 by Peters *et al.* [49] and further examined in detail by Nischang *et al.* [30,50], and Mullner *et al.* [34].

**Figure 2:**
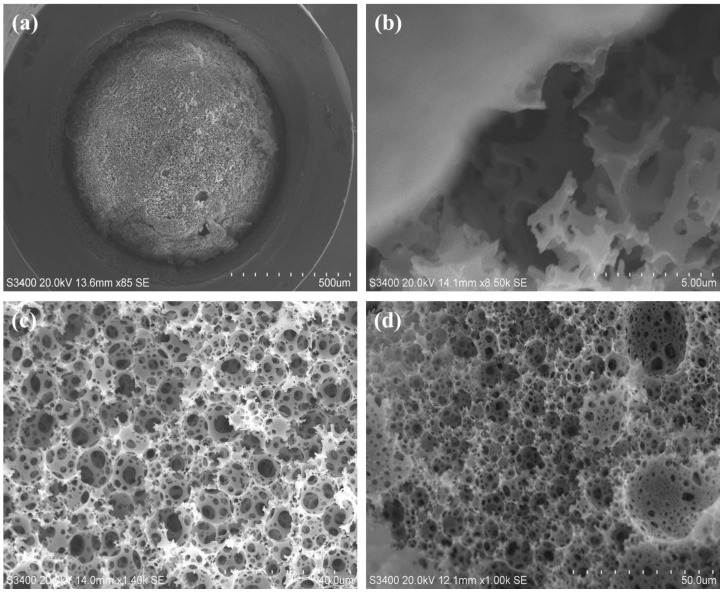
PS*-co-*DVB polyHIPE (90% porosity) formed within 1.0 mm i.d. silcosteel tubing (**a**); edge magnification of Image (**a**) showing binding to the column wall (**b**); typical polyHIPE morphology in the column centre (**c**); image of the parent, free-standing polyHIPE (**d**).

However, no such gradient requirement was necessary for the polyHIPE fabrication in 1.0 mm i.d silcosteel tubing. The polyHIPE exhibited excellent adhesion to the column housing (Column #1) as shown in Figure 2a,b. Some polyHIPE debris seen in Figure 2a was unavoidable due to cutting the tubing for SEM imaging, despite backflushing with water in attempts to remove it. Figure 2c clearly shows the characteristic voids and windows of the polyHIPE material, indicating successful polymerisation within the silcosteel tubing with no evidence of larger unwanted crater-like pores as seen in the GMA-co-EDMA polyHIPE shown in Figure 1. Void and window sizes for Column #1 were 7.7 ± 1.7 µm and 2.2 ± 1.4 µm, respectively. Interestingly, the corresponding void sizes for Batch #1 (the parent emulsion for Column #1) were notably larger at 10.3 ± 7.0 µm, and the SEM image in Figure 2d shows a number of large porous “craters”. It is theorized that the larger mould used for this free-standing monolith may have resulted in poor dissipation of the heat of polymerisation and, as such, the measured surface area for Batch #1 (20 m^2^∙g^−1^) was not considered to be indicative of the actual surface area within the smaller column housing. A second polyHIPE monolith (Column #2) was produced and the void and window sizes were found to be 49% and 45% larger relative to Column #1. This surprising difference is possibly due to the fact that only a single cross-sectional slice of each column was measured via SEM imaging multiple cross-sectional slicing of each column for comparative purposes was not considered practical. In any case, back-pressure, permeability measurements and evaluation of chromatographic performance represents a more holistic examination of the quality of the monoliths, since the entire monolith length contributes to the chromatographic performance criteria as described in the next section.

### 2.3. Backpressure and Permeability Studies for PS-co-DVB polyHIPEs

**Figure 3:**
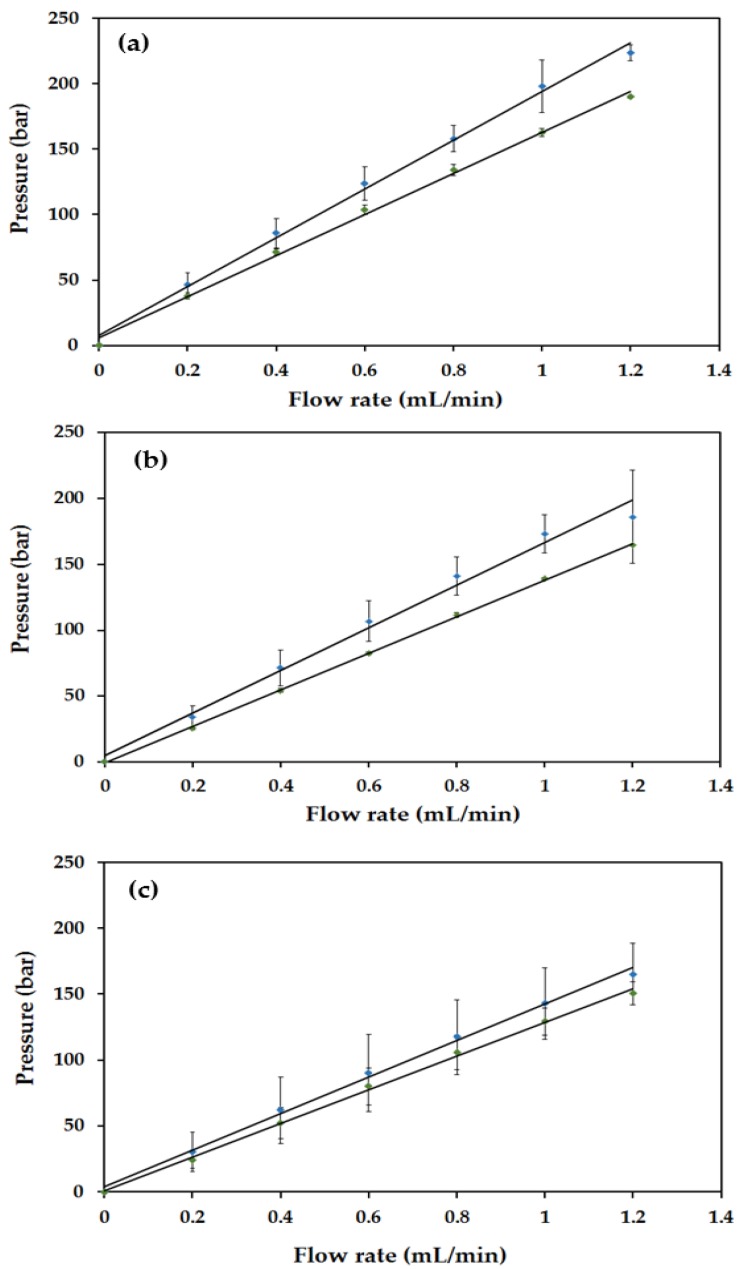
Backpressure profiles of PS*-co-*DVB polyHIPE monoliths in silcosteel tubing for water (**a**); methanol (**b**); and acetonitrile (**c**). Blue plots represent Column #1 and green plots represent Column #2. Backpressure measurements were made in triplicate at each flow rate.

Backpressure profiles were plotted for solvents commonly encountered in reversed phase HPLC (acetonitrile (ACN), MeOH and H_2_O) at flow rates of 0 to 1.2 mL/min^−1^ in 0.2 mL/min^−1^ increments. Predictably, there was a linear dependence of column pressure on flow rate, demonstrating a good mechanical stability of the monoliths, with the slopes of the backpressure plots being lowest for ACN and highest for H_2_O due to differences in solvent viscosity. However, the decrease in backpressure due to solvent viscoscity differences was not as large as expected, being only 34% from H_2_O to ACN for Column #1 despite a 62% difference in H_2_O and ACN viscosities at 25 °C. This is proposed to be due to different levels of swelling of the polymer monolith in acetonitrile. Interestingly, the backpressure for Column #1 was higher than that of Column #2 for all solvents as illustrated in Figure 3. Where permeability decreases, it is most likely due to shrinkage of the pores resulting from swelling due to the solvent used and so permeability studies using Darcy’s Law (Equation 1) were also conducted:

(1) $$k=\frac{QnL}{\Delta PA}$$

Here, *k* is the permeability coefficient (m^2^), *Q* is the flow rate (m^3^/s), *η* is the viscosity of a given solvent (Pa∙s), *ΔP* is the change in pressure (Pa), *L* is the column length (m) and *A* is the area of the column (m^2^). Typical organic polymer monoliths usually have permeabilities within the range of 10^−14^ to 10^−15^ [51,52,53]. 

The permeabilities of the PS-co-DVB polyHIPEs lay within a range of 10^−16^ to 10^−17^ and were therefore lower than literature values for more traditional polymer monoliths [52,53]; however, they were still considered acceptable, as the deviation most likely resulted from using a column of larger diameter than previously reported for capillary columns [51]. In the case of both Column #1 and Column #2, permeability was an order of magnitude lower for acetonitrile relative to water (Column #1: 1.7 × 10^−16^ for water and 8.3 × 10^−17^ for ACN; Column #2: 2.0 × 10^−16^ for water and 9.0 × 10^−17^ for ACN). It is proposed that the polyHIPE exhibited some pore shrinkage in ACN due to the wettability of the polymer (solvated polymer chains), which also accounts for the smaller than anticipated drop-off in backpressure during backpressure studies. Despite the effect of pore shrinkage observed when permeability studies were carried out, the general linear trend of the backpressure plots (Figure 3) would suggest that pore shrinkage was transient and did not permanently alter the polyHIPE morphology. 

### 2.4. Chromatographic Characterisation of PS-co-DVB PolyHIPEs

The chromatographic performance of both PS*-co-*DVB polyHIPE columns was evaluated by the separation of selected alkylbenzenes. To date, polyHIPE materials have only been demonstrated for the separation of large proteins under gradient conditions which are applications where the relatively low surface area is not detrimental to chromatographic performance. The use of polyHIPEs in CEC separations of alkylbenzenes resulted in predictably high separation efficiency (200,000 N/m) due in large part to reduced flow inhomogeneity over the cross-section of the column relative to pressure-driven flow typical of HPLC [28,29]. Stol *et al.* have demonstrated in packed-column CEC that when the pore size of stationary phase particles is large (~400 nm), a significant portion of the total flow is through the pore of the particles (previously thought to be negligible due to electrical double layer overlap) [54]. 

**Figure 4:**
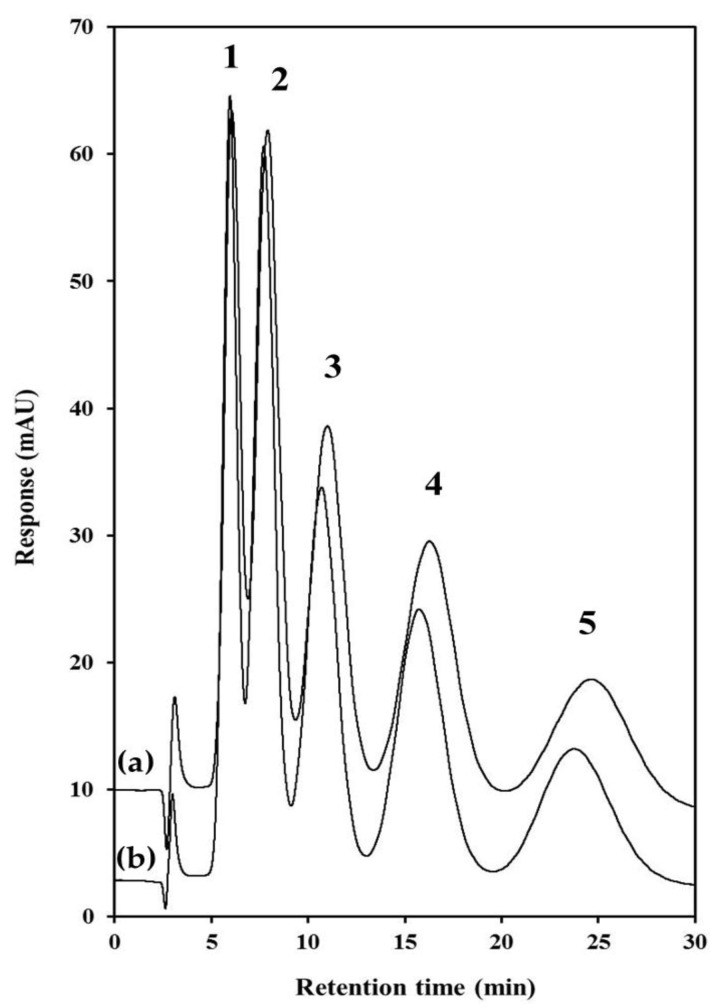
Isocratic separation of alkylbenzenes on PS-co-DVB polyHIPEs. (**a**): Column #1, (**b**): Column #2. Chromatographic conditions: Column: 1.0 mm × 100 mm 90% porosity PS-co-DVB polyHIPE, Mobile phase: 50% ACN, Flow rate: 40 µL∙min^−1^, Injection volume: 1 µL, Detection: UV at 214 nm. Peak assignments: (**1**) toluene, (**2**) ethylbenzene, (**3**) propylbenzene, (**4**) butylbenzene, (**5**) pentylbenzene. All analytes are 0.1 mg∙L^−1^.

Under these conditions, flow velocity through and between particles have roughly the same velocity and direction leading to fully perfusive behaviour and a corresponding increase in flow homogeneity across the column. Tunc *et al.* [29] reported void diameters and window diameters of 8.9 ± 2.7 and 2.0 ± 0.9 µm, respectively, which lead to fully perfusive behaviour described by Stol *et al.* [54] and much reduced stationary phase mass transfer resistance.Conversely, this report is the first instance of the isocratic separation of small molecules on a polyHIPE fabricated in a column format using pressure-driven flow. A homologous series of alkylbenzenes from toluene to pentylbenzene were well retained using a mobile phase of 50% ACN and a plot of ln(*k*) *versus* carbon number (*n_c_*) demonstrated high linearity (≥0.9995 for both columns), confirming that retention was based upon methylene group selectivity via dispersive interactions. An overlaid chromatogram for both columns is shown in Figure 4 and illustrates remarkable column-to-column reproducibility. For the most part (as shown in Table 1), retention and selectivity factors were very similar for both columns, and retention was only slightly higher for Column #2 (more noticeable for early eluters, with a retention difference of 6.1% for toluene, down to only 2.6% for pentylbenzene). The column-to-column reproducibility is all the more noteworthy given that each column was prepared on different days from separate batches of HIPE emulsion, rather than the same emulsion being filled into two separate column housings. Efficiency values were very low (3087 N/m for toluene) and decreased with increasing retention, as reported recently with commercial polymer monoliths [11,38,39].

## 3. Experimental

### 3.1. Instrumentation

All morphological characterisation was carried out using a Hitachi S-3400N scanning electron microscope (Hitachi, Maidenhead, UK) with Image J software. All samples were gold sputtered using a Quorum Technologies 750T sputter coater (Quorum Technologies, Sussex, UK). Surface area measurements were obtained using a Micromeritics Gemini 2360 surface area analyser (Micromeritics, Norcross, GA, USA). The balance used was a Sartorius Extend (Sartorius, Goettingen, Germany) and the sonication bath used was from Branson Ultrasonics Corporation (Danbury, CT, USA). A Knauer Smartline 100 V 5010 pump (Knauer, Berlin, Germany) was used for washing the prepared monoliths within silcosteel or PEEK tubing and an Ultimate 3000 capillary chromatography system (Thermo Fisher Scientific, Sunnyvale, CA, USA) was used to establish backpressure profiles for swelling studies. All van Deemter profiles and chromatographic data were acquired using an Agilent 1200 Series LC system (Agilent Technologies, Santa Clara, CA, USA). The dimensions of polyHIPE columns in silicosteel tubing was 100 mm x 1.0 mm i.d., and the mobile phase was 50:50 acetonitrile/water. The flow rate was 0.04 mL/min with an injection volume of 1 µL, column temperature was 25 °C, and UV detection was at 214 nm.

### 3.2. Materials

Ethylbenzene, propylbenzene, butylbenzene, pentylbenzene, toluene, acetone, calcium chloride dihydrate, Span-80, 3-(trimethoxysilyl) propyl methacrylate, potassium persulphate (PPS), sodium hydroxide (NaOH), hydrochloric acid, styrene and divinylbenzene were purchased from Sigma Aldrich (Sigma Aldrich, Tallaght, Ireland). Styrene and divinylbenzene were purified before use by solvent extraction with 6 %w/v NaOH to remove inhibitors. Deionised water was produced with a Millipore Direct-Q 5 (Millipore, Bedford, MA, USA). HPLC-grade methanol (MeOH) and acetonitrile (ACN) were obtained from Labscan (Labscan, Stillorgan, Dublin, Ireland). Silcosteel tubing (1.0 mm i.d.) was supplied by Restek (Restek, Belfast, UK). PEEK tubing (0.762 mm i.d.) was from Sigma Aldrich (Sigma Aldrich, Tallaght, Ireland). Alkylbenzene stock standards and working standards were prepared at 100 mg∙L^−1^ and 0.1 mg∙L^−1^ in ACN, respectively. 

#### 3.2.1. Modification of PEEK with Methacryloyl Groups

As described by Shu *et al.* [35], a solution of 50% H_2_SO_4_ was filled into PEEK tubing (1/16” o.d., 0.762 mm i.d.) and left at room temperature for six hours. The tubing was rinsed with water, filled with 1 *M* glycidyl methacrylate in acetone and incubated for four hours followed by a thorough acetone rinse and a dry-down with nitrogen.

#### 3.2.2. Silanisation of Silcosteel Tubing

Sections of silcosteel tubing (100 mm) were washed with acetone (10 min, 3 µL/min), dried with nitrogen (20 min) and then flushed sequentially with 0.2 *M* NaOH (60 min, 3 µL/min), water (10 min, 3 µL/min) and 0.2 M HCl (10 min, 3 µL/min) before further drying with a nitrogen flow for 20 min. The tubing was filled with 50% (v/v) 3-(trimethoxysilyl) propyl methacrylate in acetone, sealed and incubated at 60 °C in a water bath for 48 h. Finally, the tubing was rinsed with acetone (5 min, 3 µL/min) and dried with nitrogen.

#### 3.2.3. Fabrication of PS-co-DVB and GMA-co-EDMA PolyHIPEs

PS*-co-*DVB polyHIPEs were prepared based on a modified procedure described by Tunç *et al.* [29]. Briefly, for a 90% polyHIPE the emulsion consisted of two separately prepared phases; the aqueous phase (15 mL H_2_O, 0.03 g PPS, 0.01 g calcium chloride dihydrate) and the organic phase (1.33 mL styrene, 0.33 mL divinylbenzene, 0.33 g Span-80). The organic phase was stirred under nitrogen in a 250 mL round-bottomed flask with an overhead stirrer at 320 rpm, and the aqueous phase was added drop-wise using a hypodermic syringe. The resulting white emulsion was stirred for 20 min and then filled by syringe into silanised silcosteel tubing using an upward flow direction and sacrificial PEEK tubing at either end, to eliminate unwanted air-bubbles or voids throughout the metal housing. Columns were incubated at 60 °C for 48 h after which they were washed with MeOH at 500 µL/min for 24 h Two separate batches of 90% HIPE emulsion (Batch #1 and Batch #2) were prepared in this work and a polyHIPE column subsequently prepared from each batch, referred to hereafter as Column #1 and Column #2. Remaining emulsion not filled into column housings was polymerised as free-standing monoliths in polypropylene vials (60 °C, 48 h) followed by washing with MeOH via Soxhlet extraction for 48 h. These washed polyHIPEs were dried at 60 °C in an oven for 15 h and cut into pieces for subsequent SEM imaging and surface area analysis. (Note: during polyHIPE optimisation, a 70%, 80% and 90% polyHIPE were prepared in separate polypropylene vials and aqueous/organic phases adjusted accordingly as described above). GMA*-co-*EDMA polyHIPEs were prepared using a similar protocol except that the aqueous phase comprised (15 mL H_2_O, 0.07 g PPS, 0.5 g calcium chloride dihydrate) and the organic phase comprised (2.32 mL glycidylmethacrylate, 1.07 mL ethylene glycol dimethacrylate and 0.715 g or 0.835 g Pluronic L121), corresponding to a surfactant concentration of 4.5% or 5.5%, respectively.

#### 3.2.4. Physical Characterization of PS-*co*-DVB polyHIPEs in Batch and Column Formats

Determination of void and window size was carried out using SEM on each bulk PS*-co-*DVB polyHIPE prepared (Batch #1 and Batch #2); surface area analysis was also carried out on approximately 0.1 g of Batch #1. Backpressure and swelling tests were carried out on Column #1 and Column #2 using water, MeOH and ACN at 0.0 mL/min^−1^ to 1.2 mL/min^−1^ in 0.2 mL/min^−1^ increments. The backpressure produced at each flow rate for each solvent was recorded in triplicate. Permeability of each column was determined at 0.6 mL/min^−1^. 

## 4. Conclusions

The work presented here has shown that PS*-co-*DVB polyHIPEs with a readily tailored pore morphology can easily be formed within the confines of 1.0 mm i.d. silcosteel tubing with secure covalent attachment to the walls. Our goal was not to equal or surpass the chromatographic performance of previously reported polymer monoliths in the literature for small molecules, but rather to explore the possibility of preparing a polyHIPE material in a larger diameter column housing. To this end, this report is the first instance of a polyHIPE being formed and covalently attached to a column housing larger than 100 µm i.d. Monoliths with 90% porosity exhibiting the classical “voids and windows” bimodal pore structure could withstand pressures over 200 bar. The monolith exhibited predictable separation selectivity for alkylbenzenes, characteristic of a traditional polystyrene stationary phase. Despite the low column efficiency, it should be noted that this separation represents the first report of the isocratic separation of small molecules on a polyHIPE using pressure-driven flow. Future work will include efforts to decrease stationary phase mass-transfer resistance by optimisation of the nano-porosity of the polymer skeleton, either by the incorporation of an additional porogenic solvent in the continuous “oil” phase during polymerisation [46,55] or through the use of hyper-crosslinking using methods reported by Urban *et al.* [19.20].

## Figures and Tables

**Table 1:** Chromatographic performance criteria.

Chromatographic Performance Indicator	Toluene	Ethylbenzene	Propylbenzene	Butylbenzene	Pentylbenzene
Retention factor (*k*)	0.97 (1.03)	1.56 (1.63)	2.56 (2.65)	4.25 (4.38)	6.95 (7.13)
Selectivity factor (*α*)	1.6 (1.6)	1.6 (1.6)	1.7 (1.7)	1.6 1.6	– –
Resolution (*R_s_*)	– –	0.96 (0.98)	1.10 (1.12)	1.21 (1.25)	1.25 (1.27)
Efficiency (N/m)	2470 (3087)	2332 (2591)	1808 (2008)	1565 (1597)	1810 (1907)

Values are for Column #1, with values in parentheses representing Column #2.
